# Accuracy of oscillometric noninvasive blood pressure at the ankle in the lateral position during general anesthesia

**DOI:** 10.1186/s12871-024-02595-6

**Published:** 2024-07-03

**Authors:** Ahmed Farag, Maha Mostafa, Ahmed Hasanin, Lamis Sobh, Heba Raafat, Gihan Obayah, Maha Youssef

**Affiliations:** Department of Anesthesia and Critical Care Medicine, Faculty of Medicine, Cairo University, 01 Elsarayah street, Elmanyal, Cairo, 11559 Egypt

**Keywords:** Oscillometry, Noninvasive blood pressure, Invasive blood pressure, Lateral position, Ankle, Hypotension, Hypertension

## Abstract

**Background:**

This study aimed to evaluate the accuracy of ankle blood pressure measurements in relation to invasive blood pressure in the lateral position.

**Methods:**

This prospective observational study included adult patients scheduled for elective non-cardiac surgery under general anesthesia in the lateral position. Paired radial artery invasive and ankle noninvasive blood pressure readings were recorded in the lateral position using GE Carescape B650 monitor. The primary outcome was the ability of ankle mean arterial pressure (MAP) to detect hypotension (MAP < 70 mmHg) using area under the receiver operating characteristic curve (AUC) analysis. The secondary outcomes were the ability of ankle systolic blood pressure (SBP) to detect hypertension (SBP > 140 mmHg) as well as bias (invasive measurement – noninvasive measurement), and agreement between the two methods using the Bland-Altman analysis.

**Results:**

We analyzed 415 paired readings from 30 patients. The AUC (95% confidence interval [CI]) of ankle MAP for detecting hypotension was 0.88 (0.83–0.93). An ankle MAP of ≤ 86 mmHg had negative and positive predictive values (95% CI) of 99 (97–100)% and 21 (15–29)%, respectively, for detecting hypotension. The AUC (95% CI) of ankle SBP to detect hypertension was 0.83 (0.79–0.86) with negative and positive predictive values (95% CI) of 95 (92–97)% and 36 (26–46)%, respectively, at a cutoff value of > 144 mmHg. The mean bias between the two methods was − 12 ± 17, 3 ± 12, and − 1 ± 11 mmHg for the SBP, diastolic blood pressure, and MAP, respectively.

**Conclusion:**

In patients under general anesthesia in the lateral position, ankle blood pressure measurements are not interchangeable with the corresponding invasive measurements. However, an ankle MAP > 86 mmHg can exclude hypotension with 99% accuracy, and an ankle SBP < 144 mmHg can exclude hypertension with 95% accuracy.

## Introduction

Arterial blood pressure monitoring is standard care during anesthesia. Maintaining normal blood pressure and preventing hypotension are fundamental rules for anesthetists. Intraoperative hypotension is associated with increased risk of postoperative morbidity and mortality [1]. The hypotension threshold varies across studies; however, mean arterial pressure (MAP) ranges of < 60–70 mmHg are the most acceptable [1]. Furthermore, to maintain adequate intraoperative blood pressure, accurate measurement of arterial blood pressure is needed.

Noninvasive arm blood pressure measurement is the most commonly used method in daily practice. To achieve accurate noninvasive blood pressure measurements, the blood pressure cuff should be at the level of the heart [2]; however, maintaining this is not possible when the patient is in the lateral position. Many surgical procedures are performed in the lateral position, with some, such as orthopedic and plastic procedures, performed on the upper extremity. Therefore, the accessibility of the upper arm for placement of the blood pressure cuff is limited. Furthermore, noninvasive arm blood pressure measurements have been reported to be inaccurate in the lateral position [3]. Therefore, alternative sites should be evaluated, particularly when invasive measurements are not possible or are inconvenient.

The ankle is the most accessible site and can be easily adjusted to the level of the heart in the lateral position. Previous data assessing ankle blood pressure in relation to invasive measurements were obtained in the supine position [4]. However, to the best of our knowledge, there is no data regarding the accuracy of ankle blood pressure in terms of detecting hemodynamic instability (hypotension and/or hypertension) in patients under general anesthesia in the lateral position.

The main objective of this study was to evaluate the ability of ankle blood pressure to detect intraoperative hypotension in the lateral position, using invasive blood pressure as a reference. The secondary objectives were to evaluate its ability to detect hypertension, as well as its accuracy and trending ability in relation to invasive measurements in the lateral position.

## Methods

This prospective observational study was conducted at Cairo University Hospital surgical theatre between May 2022 and July 2023, after obtaining institutional ethics committee approval (MD-359-2022). Written informed consent was obtained from all patients before enrollment.

The participants were adult patients (> 18 years) with an American Society of Anesthesiologist-physical status (ASA-PS) of I–III who were scheduled for elective non-cardiac surgery under general anesthesia in the lateral position and required invasive blood pressure monitoring.

The exclusion criteria were the presence of arrhythmia, peripheral vascular diseases, deep venous thrombosis, lower limb edema, scarring, previous ankle fractures or surgeries, and body mass index > 35 kg/m^2^.

Before anesthesia induction, all patients were monitored using noninvasive blood pressure measurement at the arm, a five-lead electrocardiogram, and pulse oximetry.

After anesthesia induction, a 20-G radial arterial catheter was inserted. The arterial catheter was connected to a pressure transducer placed at the level of mid-axillary line when the patient was in the supine position and at the mid-sternum when the patient was in the lateral position.

An appropriately-sized noninvasive blood pressure cuff (CRITIKON DURA-CUF®, GE HealthCare, Germany) for the ankle was selected based on the ankle circumference just above the malleoli and according to the American Heart Association recommendation (cuff length and width should be 80% and 40% of arm circumference, respectively). In the lateral position, ankle level was maintained at heart level using supporting pillows. The monitor used in this study was a GE Carescape B650 (GE HealthCare, Germany).

Paired invasive and noninvasive ankle blood pressure readings were recorded while the patient was in the lateral position. Invasive blood pressure readings were recorded during ankle cuff inflation.

For evaluation of the trending ability, the change in invasive and noninvasive blood pressure readings (Δ) was calculated as the difference between two consecutive readings.

Intraoperative hemodynamic management was based on the discretion of the attending anesthetist, which mainly involved administration of a fluid bolus or vasopressors in case of hypotension, deepening the anesthesia, or administration of a vasodilator in case of hypertension.

The primary outcome of the study was the ability of the ankle MAP to detect an invasive MAP < 70 mmHg while in the lateral position.

Secondary outcomes were the ability of ankle systolic blood pressure (SBP) to detect invasive SBP > 140 mmHg, bias (invasive measurement – noninvasive measurement) and agreement between the two methods, number of paired readings with a difference of ≤ 5, 10, and 15 mmHg, and the trending ability of ankle blood pressure in relation to the invasive blood pressure.

### Sample size

The sample size was calculated using MedCalc Software version 14 (MedCalc Software bvba, Ostend, Belgium) to detect an area under the receiver operating characteristic curve (AUC) of 0.75 with a null hypothesis AUC of 0.5. Considering that the number of hypotensive readings would be 5% of the total readings, we calculated a minimum of 300 pairs of readings (with at least 15 hypotensive readings) for a study power of 90% and an alpha error of 0.05. These readings were expected to be obtained from a minimum of 30 patients (at least 10 readings per patient).

### Statistical analysis

Statistical package for social science version 26 for Microsoft Windows (IBM Corp., NY, USA), SAS ONDEMAND FOR ACADEMICS (Copyright © 2012–2020, SAS Institute Inc., Cary, NC, USA), and MedCalc software were used for data analysis. Data were tested for normality using the Shapiro-Wilk test. Data are presented as mean ± standard deviation or median (quartiles) according to the data distribution. The ability of ankle blood pressure to detect hypotension and hypertension was assessed using the AUC analysis adjusted for repeated measurements. The Youden index was used to identify the optimal cutoff value. Statistical significance was set at P-value < 0.05. The Bland-Altman analysis adjusted for multiple readings per subject was performed to assess the mean bias and 95% agreement between the two methods [5]. A four-quadrant plot with a central exclusion zone of < 5 mmHg was used to assess the trending ability of ankle blood pressure in relation to invasive blood pressure. The concordance rate was calculated as the ratio of the number of points in the upper-right and lower-left quadrants to the total number of points in all four quadrants.

## Results

**Fig. 1:**
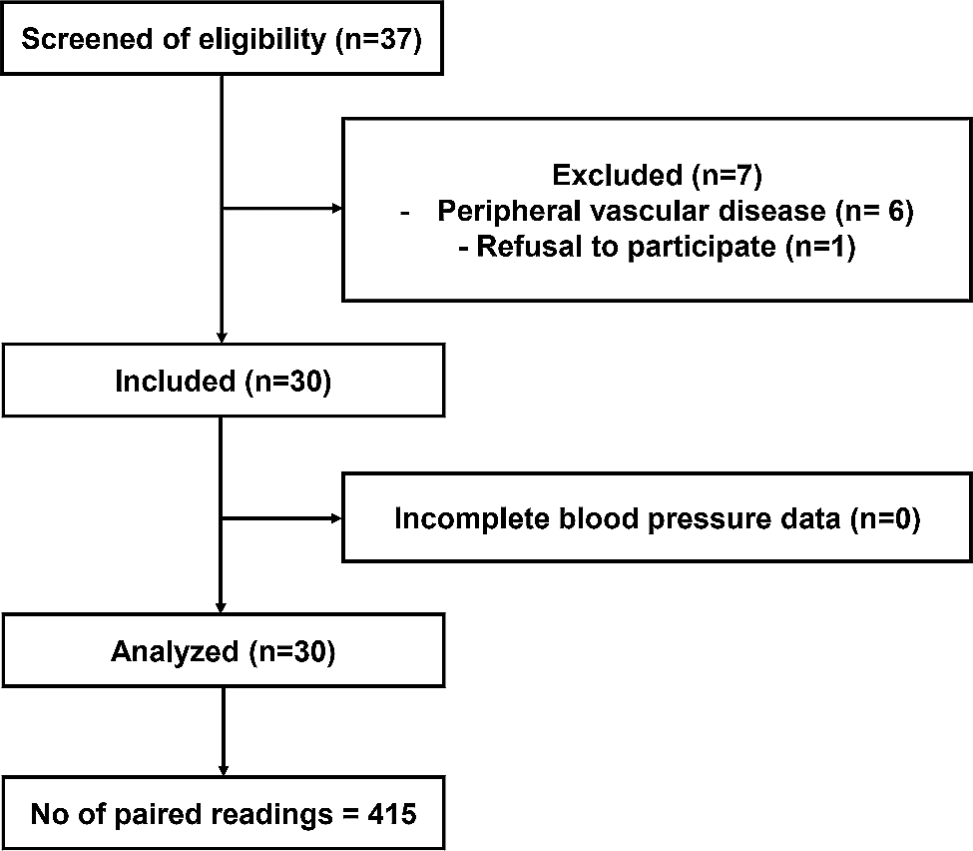
Patients’ enrollment flowchart

Thirty-seven patients were screened for eligibility, and seven were excluded because they did not fulfill the inclusion criteria. Thirty patients were included, of whom 415 paired invasive and ankle blood pressure readings were obtained and analyzed. (Fig. 1)

The demographic and hemodynamic data are presented in Table 1.

**Table 1:** Demographic data. Data are presented as mean ± standard deviation and frequency (%)

	*N* = 30
Age (years)	41 ± 10
Male sex	19 (63%)
Weight (kg)	82 ± 12
Height (cm)	170 ± 6
Body mass index (kg/m^2^)	28 ± 4
Ankle circumference (cm)	24 ± 4
Procedure, n (%)	
Urological surgery Orthopedic surgery Thoracoscopy	18 (60%) 7 (23%) 5 (17%)
Invasive blood pressure, mean ± SD (minimum, maximum) (mmHg)	
SBP DBP MAP	117 ± 19 (70, 167) 75 ± 13 (42, 110) 91 ± 15 (50, 129)

DBP: diastolic blood pressure, MAP: mean arterial pressure, SBP: systolic blood pressure

**Fig. 2:**
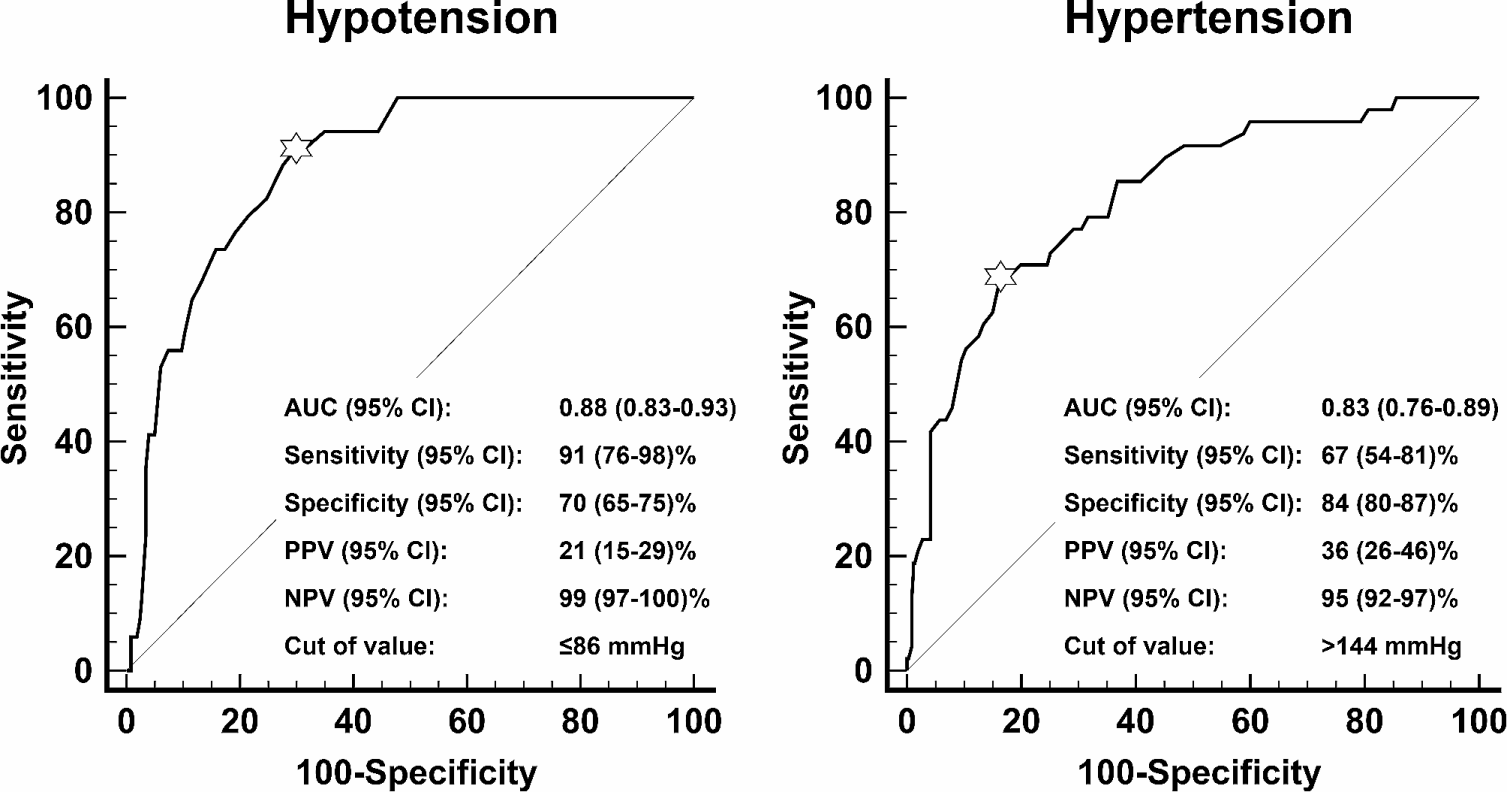
Receiver operating characteristics for the ability of ankle MAP to detect hypotension (left) and ankle SBP to detect hypertension (right). The mark indicates the cutoff value that corresponds to the Youden index. CI: confidence interval, DBP: diastolic blood pressure, MAP: mean arterial pressure, NPV: negative predictive value, PPV: positive predictive value, SBP: systolic blood pressure

Eleven patients developed intraoperative hypotension and 34 hypotensive readings were collected in total. The AUC (95% confidence interval) for ankle MAP for detecting hypotension was 0.88 (0.83–0.93). An ankle MAP of ≤ 86 mmHg had a negative predictive value of 99% for detecting hypotension. (Fig. 2)

Forty-eight hypertensive readings were obtained. The AUC (95% confidence interval) for ankle SBP to detect hypertension was 0.83 (0.76–0.89) with a negative predictive value of 95% at a cutoff value of > 144 mmHg. (Fig. 2)

**Fig. 3:**
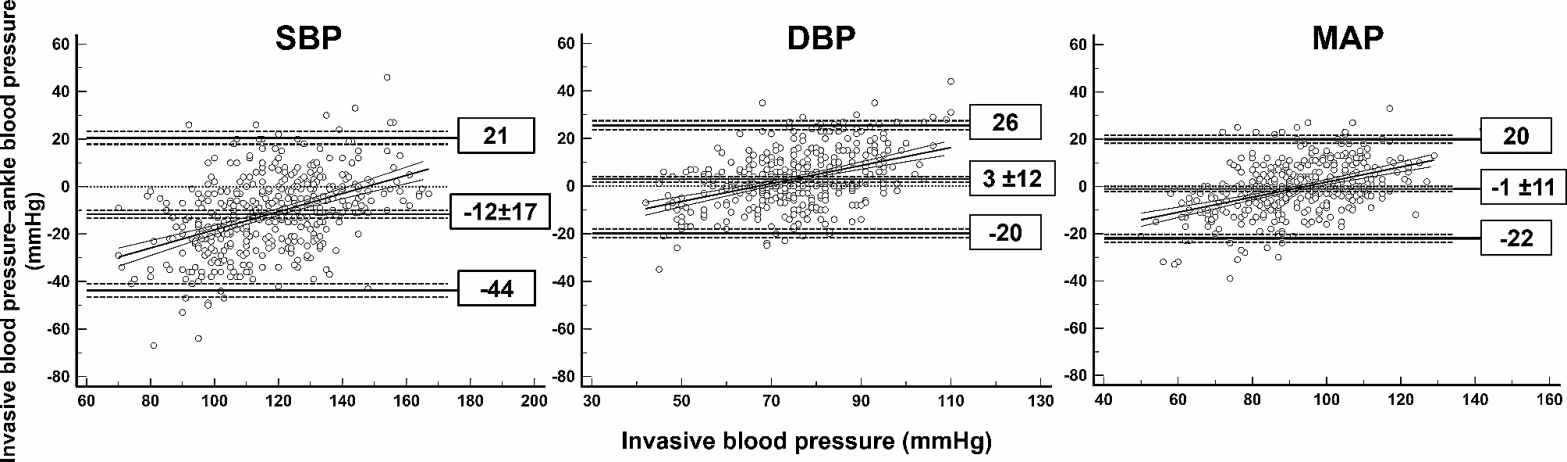
Bland-Altman plot. The horizontal solid lines are the mean bias and its 95% limits of agreement. The dashed lines are the 95% confidence interval of the limits of agreement. The oblique line represents the regression line linking the mean bias between the two methods and invasive blood pressure. DBP: diastolic blood pressure, MAP: mean arterial pressure, SBP: systolic blood pressure

The mean bias between the invasive and noninvasive measurements was − 12 ± 17, 3 ± 12, and − 1 ± 11 mmHg for the SBP, diastolic blood pressure (DBP), and MAP, respectively. (Fig. 3)

The number of paired readings with difference ≤ 15 mmHg was 232/415 (56%), 321/415 (77%), 363/415 (88%) for the SBP, DBP, and MAP, respectively. (Table 2)

**Table 2:** Number of paired readings with absolute difference of ≤ 5, 10, 15 mmHg. Data are presented as frequency (%)

	≤ 5mmHg	≤ 10mmHg	≤ 15mmHg
SBP	91 (22%)	171 (41%)	232 (56%)
DBP	174 (42%)	278 (67%)	321 (77%)
MAP	164 (40%)	290 (70%)	363 (88%)

DBP: diastolic blood pressure, MAP: mean arterial pressure, SBP: systolic blood pressure

**Fig. 4:**
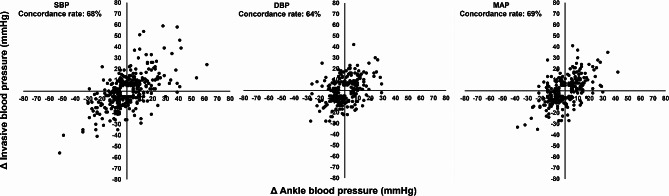
Four-quadrant scatter plot for the trending values of the invasive and ankle blood pressure measurements. A central exclusion zone of < 5 mmHg was used. DBP: diastolic blood pressure, MAP: mean arterial pressure, SBP: systolic blood pressure

Regarding the trending ability of ankle blood pressure in relation to invasive measurements, the concordance rates between the two methods were 68%, 64%, and 69% for the SBP, DBP, and MAP, respectively. (Fig. 4)

## Discussion

In this study, we evaluated the ability of ankle blood pressure to detect hypotension and hypertension as well as its accuracy in relation to invasive blood pressure measurement. Our results revealed that ankle MAP showed a good ability to exclude hypotension (MAP < 70 mmHg). An ankle MAP ≤ 86 mmHg had a negative predictive value of 99% and a positive predictive value of 21%. Therefore, an ankle MAP of > 86 mmHg can accurately rule out hypotension (high negative predictive value); however, an ankle MAP of ≤ 86 mmHg cannot confirm the presence of hypotension (low positive predictive value). Furthermore, ankle SBP < 144 mmHg had good ability to exclude hypertension, with a negative predictive value of 95%.

To the best of our knowledge, this is the first study to evaluate ankle blood pressure in the lateral position using invasive blood pressure as a reference standard. The ability of ankle blood pressure to detect hypotension was previously evaluated by Lakhal et al. [4] in critically ill patients. The authors reported an AUC of 0.90 for ankle MAP to detect hypotension, which is close to that reported in the current study (0.88) [4]. However, our study differs from the study by Lakhal et al. [4] in that we included patients under general anesthesia in the lateral position, while Lakhal et al. included mostly patients with circulatory shock who were already on vasopressor therapy and assessed ankle blood pressure in the supine position. Furthermore, we assessed the ability of ankle blood pressure to detect hypertension and its trending ability.

According to the American National Standards Institute/Association for the advancement of Medical Instrumentation/International Organization for Standardization (ANSI/AAMI/ISO) standard, the mean difference ± standard deviation between the two methods should be < 5 ± 8 mmHg [6]. In this study, the mean bias between the two methods was − 12 ± 17, 3 ± 12, and − 1 ± 11 mmHg for the SBP, DBP, and MAP, respectively, which is higher than the defined limits to meet the ANSI/AAMI/ISO standards in all blood pressure components. Furthermore, the number of paired readings with difference of ≤ 5, 10, 15 mmHg was lower than the minimum requirement for the European Society of Hypertension (ESH) standards (at least 65, 81, and 93% of the differences between the two methods falling within 5-, 10-, and 15-mm Hg, respectively) [7]. Therefore, ankle blood pressure did not fulfil the ESH standard for all blood pressure components. A concordance rate of > 90% indicates a good trending ability. In this study, the trending ability of ankle blood pressure was poor (concordance rate < 90% for all blood pressure components [8]).

Our study showed that ankle blood pressure has poor accuracy and is not interchangeable with invasive pressure, which is supported by previous studies in the supine position in critically ill adults [4, 9], patients with obesity [10], and pediatric patients [11]. Several factors could explain this inaccuracy. First, current oscillometric devices and noninvasive cuffs have been developed and validated for arm blood pressure measurements, but not for the ankle. Second, ankle blood pressure measurement is obtained by compressing the posterior tibial artery against two bones, which might reduce the accuracy of the measurement, unlike compressing against one bone, as in arm measurements. Third, the pulse pressure amplification phenomenon (the increase in SBP and decrease in DBP in the distal arteries in relation to the more proximal arteries) could be another contributing factor for this inaccuracy [12].

Invasive blood pressure measurement is the gold standard for measuring blood pressure. However, this method, being invasive and expensive, is only reserved for patients requiring precise and immediate hemodynamic management. Therefore, oscillometric blood pressure devices are standard monitors for blood pressure measurements in patients under anesthesia [13]. The arm is the standard site for noninvasive blood pressure measurement [13]. However, using the arm to measure blood pressure in the lateral position has several limitations in terms of accuracy and feasibility. Mostafa et al. [3] reported that neither the dependent nor the non-dependent arms were accurate for blood pressure measurement in the lateral position, and neither was interchangeable with the invasive measurement. The position of the arm in relation to the heart in the lateral position could explain this inaccuracy. In addition, there are several surgical procedures in which the ankle is the only available site for blood pressure monitoring, and invasive blood pressure monitoring is inconvenient, especially for minor procedures. These limitations in the use of the arm for blood pressure measurement in the lateral position highlight the need for alternatives. The ankle is an appealing alternative to the arm in such position, especially because the ankle, unlike the arm, can be maintained at heart level in the lateral position, which might improve its accuracy. The results of this study could not validate ankle measurements in terms of absolute or trending values. However, the results of this study showed that the ankle can be pragmatically used to identify hemodynamic instability (hypotension and hypertension), which is the main objective of blood pressure monitoring during anesthesia, regardless of the accuracy of absolute values. The results of this study suggest that an ankle MAP > 86 mmHg can accurately exclude hypotension, and an ankle SBP < 144 mmHg can exclude hypertension.

This study had some limitations. This was a single-center study. We included patients without peripheral vascular disease, arrhythmia, or obesity; therefore, our results are limited to the included population, and further studies are required in other populations. A single model of an oscillometric device and a noninvasive blood pressure cuff were evaluated; therefore, the results of this study are limited to the device used. We derived our data from a cohort of 30 patients; therefore, further studies are needed to validate our findings. Hypotension is generally undesirable during anesthesia due to its harmful impact on patient outcomes [1]. Thus, our practice includes several strategies to avoid and treat hypotension aggressively. This might explain the low frequency of hypotensive episodes in the study. However, this point was carefully considered during the sample size calculation.

## Conclusion

In patients under general anesthesia in the lateral position, ankle blood pressure measurements are not interchangeable with the corresponding invasive measurements and have a poor trending ability. However, an ankle MAP > 86 mmHg can exclude hypotension with 99% accuracy, and an ankle SBP < 144 mmHg can exclude hypertension with 95% accuracy.

## Data Availability

The datasets used and/or analyzed during the current study are available from the corresponding author upon reasonable request.
